# Supplementary material for: Extracting causation from millennial-scale climate fluctuations in the last 800 kyr
Source: Sci Rep. 2022 Sep 12;12:15320. doi: 10.1038/s41598-022-18406-2 (PMC9468010; doi:10.1038/s41598-022-18406-2)
Supplement: Supplementary file 1 — Supplementary Information. [file 41598_2022_18406_MOESM1_ESM.pdf]

Supplemental material: Extracting causation from millennial-scale climate fluctuations in the last 800 kyr

Marco Baldovin[1,4,*], Fabio Cecconi[2], Antonello Provenzale[3], and Angelo Vulpiani[4]

[1]Université Paris-Saclay, CNRS, LPTMS, 91405, Orsay, France
[2]CNR-Istituto dei Sistemi Complessi, Rome, I-00185, Italy
[3]CNR-Istituto di Geoscienze e Georisorse, Pisa, I-56124 Italy
[4]Dipartimento di Fisica, Università "Sapienza", Rome I-00185, Italy
[*]marco.baldovin@universite-paris-saclay.fr

ABSTRACT

The supplemental material presented here aims at providing further information about our research, in particular about the analytical results used in the main text and an analysis of the possible issues related to the non-homogeneous density of the available data along the considered time interval. In what follows, the reader can find:
1) a complete derivation of the generalized Fluctuation Dissipation Relation;
2) the estimation of the error associated to the filtered procedure discussed in the main text;
3) a series of numerical examples which illustrate the method (and show its robustness against the inclusion of small nonlinear terms in the fast dynamics);
4) explicit formulas for the transfer entropy in the linear case;
5) further methodological remarks for the application of our method to paleoclimate datasets.

Derivation of the generalized FDR

In this section we briefly sketch the derivation of formula (2) of the main text. A more detailed exposition can be found in Ref.[1]. Let $\mathbf{x}(t) = [x_1(t), \ldots, x_n(t)]$ be a multivariate Markov process of dimension n, whose stationary PDF $p_{st}(\mathbf{x})$ is smooth and nonvanishing. We want to understand the effect on the dynamics $\mathbf{x}(t)$ of an instantaneous small perturbation $\varepsilon = (\varepsilon_1, \ldots, \varepsilon_n)$ performed at time $t = 0$, by measuring the displacement it generates on the averages

$$\delta\langle \mathbf{x}(t)\rangle = \langle \mathbf{x}(t)\rangle_\varepsilon - \langle \mathbf{x}(t)\rangle_0 \,, \tag{S1}$$

where the first term corresponds to the perturbed dynamics and the second to the original (unperturbed) one. The average should be interpreted as carried out over many realizations of the perturbed and the original dynamics. The analytical computation of Eq.(S1) requires the knowledge of the joint probabilities of $\mathbf{x}(t)$ and $\mathbf{x}_0$, that for a Markov process can be expressed as

$$P[\mathbf{x}(t), \mathbf{x}_0] = p_{st}(\mathbf{x}_0)\, W(\mathbf{x}, t\,|\,\mathbf{x}_0) \tag{S2}$$

$$P_\varepsilon[\mathbf{x}(t), \mathbf{x}_0] = p_\varepsilon(\mathbf{x}_0)\, W(\mathbf{x}, t\,|\,\mathbf{x}_0) = p_{st}(\mathbf{x}_0 - \varepsilon)\, W(\mathbf{x}, t\,|\,\mathbf{x}_0) \tag{S3}$$

where as the perturbation involves only the initial condition, the probability density of the perturbed system is nothing but a rigid shift of the invariant distribution of the unperturbed system; for example, in the scalar case if $p_{st}(x_0) = 1/\sqrt{2\pi\sigma^2}\exp[-x^2/(2\sigma^2)]$ is a Gaussian with zero average, the perturbation will bring the system in a Gaussian distribution with average ε, i.e. $1/\sqrt{2\pi\sigma^2}\exp[-(x-\varepsilon)^2/(2\sigma^2)]$. Moreover, because the perturbation affects only initial states, the evolution rule in unchanged thus both systems will share the same transition probability (propagator) $W(\mathbf{x}, t\,|\,\mathbf{x}_0)$, see Ref.[1]. So we can write,

$$\delta\langle \mathbf{x}(t)\rangle = \int d\mathbf{x}_0 \int d\mathbf{x}\, \frac{p_{st}(\mathbf{x}_0 - \varepsilon) - p_{st}(\mathbf{x}_0)}{p_{st}(\mathbf{x}_0)}\, p_{st}(\mathbf{x}_0)\, \mathbf{x}\, W_t(\mathbf{x}, t|\mathbf{x}_0) \,.$$

Since $|\varepsilon| \ll 1$, the above expression can be expanded to the first order in $|\varepsilon|$, leading to the formula

$$\delta\langle \mathbf{x}_i(t)\rangle = -\sum_{j=1}^{n} \varepsilon_j \left\langle x_i \frac{\partial \ln p_{st}(\mathbf{x})}{\partial x_j}\bigg|_0 \right\rangle.$$

Then the response function of the generalized (FDR) reads

$$R_{ij}(t) = -\left\langle x_i(t) \frac{\partial \ln p_{st}(\mathbf{x})}{\partial x_j}\Big|_{\mathbf{x}(0)} \right\rangle, \tag{S4}$$

where the average $\langle\cdot\rangle$ is computed on the unperturbed system, and R_t is the matrix of the linear response functions. The above equation is valid if the system admits a (sufficiently smooth) invariant distribution, $p_{st}(\mathbf{x})$.

When the response formula (S4) is applied to a linear Markov process, namely a Ornstein-Uhlenbeck process (OUP), we obtain exactly Eq.(3) of the main text. The OUP is defined by the evolution

$$\frac{d\mathbf{x}}{dx} = \mathbb{A}\mathbf{x}_t + \mathbb{B}\eta(t) \tag{S5}$$

where $\mathbb{A}$ and $\mathbb{B}$ are constant $n \times n$ matrices, and $\eta(t)$ is an array of n independent delta-correlated Gaussian noises. The stationary PDF of the OUP, that is required in Eq.(S4), is known to be[2]

$$p_s(\mathbf{x}) = \frac{1}{\sqrt{\mathrm{Det}(2\pi\Sigma)}} \exp\left\{ -\frac{1}{2}\mathbf{x}^T\Sigma^{-1}\mathbf{x} \right\}$$

where $\Sigma = \mathrm{var}(\mathbf{x}) = \langle \mathbf{x}\mathbf{x}^T \rangle$ is the stationary variance, that for the OUP is

$$\Sigma = \int_0^\infty dt e^{-\mathbb{A}t}\mathbb{B}\mathbb{B}^T e^{-\mathbb{A}^T t},$$

thus independent of time. Now by applying Eq.(S4), we straightforwardly obtain

$$\mathbb{R}(t) = \mathbb{C}(t)\Sigma^{-1} \tag{S6}$$

that is exactly the formula (3) of the main text, upon realizing that $\Sigma = \mathbb{C}(0)$.

Eq. (S4) expresses the link among responses and correlators of every orders, it allows the response functions to be computed from suitable correlation functions, provided that the functional form of $P_s(\mathbf{x})$ is known either a priori or inferred from data (often a completely non-trivial task).

Evaluation of the error

In this section we provide an esteem of the error associated with the filtering procedure discussed in the main text. In short, we compute the correlation functions of the filtered process showing that they are equal to those of the fast variables defined in the Methods section, but for an additive constant which depends on the time scale separation between the fast dynamics, the slow dynamics and the filtering window T_w.

We start by noticing that Eq. (12) of the main text implies

$$\tilde{\mathbf{x}}(t) = \mathbf{x}_S(t) + \mathbf{x}_F(t) - \int_{-\infty}^{\infty} ds \mathscr{G}(t-s)\mathbf{x}_S(s) - \int_{-\infty}^{\infty} ds \mathscr{G}(t-s)\mathbf{x}_F(s)\,. \tag{S7}$$

The slow-variable evolution is ruled by Eq. (7) of the main text, whose complete solution is

$$\mathbf{x}_S(t) = e^{-\mathbb{A}t}\mathbf{x}_S(0) + \int_0^t ds\, e^{-\mathbb{A}(t-s)}\mathbf{c}f(s)\,. \tag{S8}$$

The first term on the right hand side is a transient, whose contribution becomes negligible as soon as t is larger than a few τ_0. To evaluate the integral, let us remember that $f(t)$ is a slow-varying function, so that $f(s) \simeq f(t) - f'(t)(t-s)$ with $f'(t) \simeq O(\tau_1^{-1})$. We get therefore

$$\mathbf{x}_S(t) \simeq \mathbb{A}^{-1}\mathbf{c}f(t) + O(\tau_0/\tau_1)\,; \tag{S9}$$

as a consequence, the third term of the right hand side of Eq. (S7) reads

$$\begin{aligned}\int_{-\infty}^{\infty} ds \mathscr{G}(t-s)\mathbf{x}_S(s) &\simeq \mathbb{A}^{-1}\mathbf{c}\sum_{j=1}^{l} a_j \int_{-\infty}^{\infty} ds \mathscr{G}(t-s)\cos(s/\tau_j + \phi_j) + O(\tau_0/\tau_1) \\ &\simeq \mathbb{A}^{-1}\mathbf{c}\sum_{j=1}^{l} a_j \cos(t/\tau_j + \phi_j) e^{-T_w^2/2\tau_j^2} + O(\tau_0/\tau_1) \\ &\simeq \mathbf{x}_S + O(\max\{T_w^2/\tau_1^2, \tau_0/\tau_1\})\,,\end{aligned} \tag{S10}$$

where also Eq. (5) of the main text has been exploited. The filtered variables can thus be approximated as

$$\tilde{\mathbf{x}}(t) \simeq \mathbf{x}_F(t) - \int_{-\infty}^{\infty} ds \mathscr{G}(t-s)\mathbf{x}_F(s) + O(\max\{T_w^2/\tau_1^2, \tau_0/\tau_1\}) \,. \tag{S11}$$

We want to estimate the correlation functions appearing in the generalized FDR for linear dynamics. In the light of the above, one has

$$\langle \tilde{\mathbf{x}}(t)\tilde{\mathbf{x}}^T(t')\rangle \simeq \left\langle \left(\mathbf{x}_F(t) - \int_{-\infty}^{\infty} ds \mathscr{G}(t-s)\mathbf{x}_F(s)\right)\left(\mathbf{x}_F(t') - \int_{-\infty}^{\infty} ds \mathscr{G}(t'-s)\mathbf{x}_F(s)\right)^T \right\rangle + O(\max\{T_w^2/\tau_1^2, \tau_0/\tau_1\}) \,. \tag{S12}$$

The product in the average in the r.h.s. leads to four terms, one of which is $\langle \mathbf{x}_F(t)\mathbf{x}_F^T(t')\rangle$. We have to show that the remaining ones are negligible.

To estimate these terms it is important to remind the properties of the matrix $\mathbb{A}$ and the fact that the dynamics $\mathbf{x}_F$ is given by Eq. (8) of the main text, so that[2]

$$\langle \mathbf{x}_F(t)\mathbf{x}_F^T(t')\rangle = \begin{cases} e^{-\mathbb{A}(t-t')}\Sigma & \text{if} \quad t > t' \\ \Sigma e^{-\mathbb{A}^T(t'-t)} & \text{if} \quad t < t' \end{cases} \tag{S13}$$

where Σ is the covariance matrix $\Sigma = \langle \mathbf{x}_F(t)\mathbf{x}_F^T(t)\rangle$.

$$\begin{aligned} \left\langle \mathbf{x}_F(t) \int_{-\infty}^{\infty} ds \mathscr{G}(t'-s)\mathbf{x}_F^T(s) \right\rangle &= \int_{-\infty}^{t} ds \mathscr{G}(t'-s) e^{-\mathbb{A}(t-s)}\Sigma + \Sigma \int_{t}^{\infty} ds \mathscr{G}(t'-s) e^{-\mathbb{A}^T(t'-t)} \\ &= \mathbb{V}^{-1} \int_{-\infty}^{t} ds \mathscr{G}(t'-s) e^{-\mathbb{A}_D(t-s)} \mathbb{V}\Sigma + \Sigma \mathbb{U}^{-1} \int_{t}^{\infty} ds \mathscr{G}(t'-s) e^{-\mathbb{A}_D^T(t'-t)} U \end{aligned} \tag{S14}$$

where

- $\mathbb{V}$ is the matrix that diagonalizes $\mathbb{A}$;
- $\mathbb{U} = (\mathbb{V}^T)^{-1}$ is the matrix diagonalizing $\mathbb{A}^T$;
- $\mathbb{A}_D$ is the corresponding diagonal matrix: $\mathbb{A} = \mathbb{V}^{-1}\mathbb{A}_{\mathbb{D}}\mathbb{V}$, $\mathbb{A}^T = \mathbb{U}^{-1}\mathbb{A}_D U$.

In the above reasoning we have exploited our hypothesis about the diagonalizability of the matrix A. This condition simplifies the computation, as it allows to write the exponential of the matrix in a simple way. Let us stress, however, that a similar calculation could be also carried out for generic invertible matrixes, by making use of the Jordan normal form to compute the exponentials. The above integrals are now diagonal matrices, and we can evaluate the j-th diagonal element as

$$\int_{-\infty}^{t} ds \mathscr{G}(t'-s) e^{-a_D^j(t-s)} = \frac{1}{2} e^{a_D^j(t'-t) + (a_D^j)^2 T_w^2/2} \text{erfc}\left[\frac{t'-t+a_D^j T_w^2}{\sqrt{2}T_w}\right] \simeq \frac{e^{-(t'-t)^2/2T_w^2}}{\sqrt{2\pi}a_D^j T_w} \tag{S15a}$$

$$\int_{t}^{\infty} ds \mathscr{G}(t'-s) e^{-a_D^j(t'-t)} = \frac{1}{2} e^{a_D^j(t-t') + (a_D^j)^2 T_w^2/2} \text{erfc}\left[\frac{t-t'+a_D^j T_w^2}{\sqrt{2}T_w}\right] \simeq \frac{e^{-(t'-t)^2/2T_w^2}}{\sqrt{2\pi}a_D^j T_w} \tag{S15b}$$

where a_D^j is the j-th diagonal element of the matrix A_D, and we have exploited the asymptotic expansion $\text{erfc}(x) \simeq e^{-x^2}/(x\sqrt{\pi})$, valid for $x \gg 1$. Since we are indeed interested in the time-range in which $t-t'$ is at most $O(T_w)$, and recalling that the spectral radius of A is order τ_0^{-1}, we can conclude that the above terms are at most $O(\tau_0/T_w)$. As a consequence,

$$\left\langle \mathbf{x}_F(t) \int_{-\infty}^{\infty} ds \mathscr{G}(t'-s)\mathbf{x}_F^T(s) \right\rangle \simeq O(\tau_0/T_w) \,. \tag{S16}$$

Reasoning in the same way for the remaining terms one obtains

$$\langle \tilde{\mathbf{x}}(t)\tilde{\mathbf{x}}^T(t')\rangle \simeq \langle \mathbf{x}_F(t)\mathbf{x}_F^T(t')\rangle + O(\max\{\tau_0/T_w, T_w^2/\tau_0^2\}) \,, \tag{S17}$$

since terms $O(\tau_0/\tau_1)$ are always negligible w.r.t. $O(\tau_0/T_w)$.

The sources of error in the approximation are therefore two. To minimize the error, one has to choose T_w of the order of $T_w^\star = (\tau_0\tau_1^2)^{1/3}$.

Numerical examples

To illustrate how the above proposed combination of filtering and GFDR works in practice, we consider some pedagogical examples based on suitably defined toy models.

A toy model with linear fast dynamics

Let $\mathbf{x}$ be the two-dimensional system defined by Eq. (4) of the main text, where A is the 2×2 matrix

$$\mathbb{A} = \frac{1}{\tau_0}\begin{pmatrix} 1 & a_{12} \\ a_{21} & 1 \end{pmatrix}, \qquad \text{(S18)}$$

and the forcing reads

$$f(t) = \cos\left(\frac{2\pi t}{\tau_1(1+\varepsilon)}\right) + \cos\left(\frac{2\pi t}{\tau_1(1-\varepsilon)}\right). \qquad \text{(S19)}$$

Finally, $\mathbf{c} = (1/2, 1/2)$ and $D = 1/\tau_0$. In this case the fastest characteristic time of the slow dynamics is $O(\tau_1)$, while the typical time-scale of the fast one is τ_0.

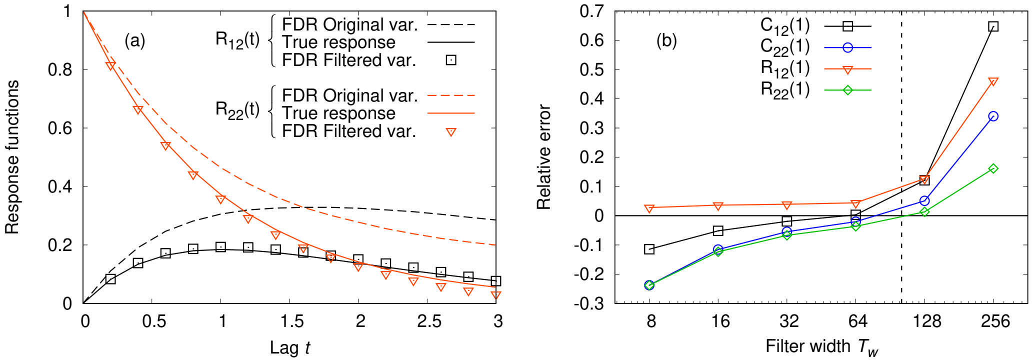

Figure S1. Toy model with forcing, Eq. (S19). Panel (a) shows two response functions, measured from numerical simulations (solid lines), compared to the GFDR obtained with the filtered variables ($T_w = 64$, points) and with the original variables (dashed lines). Panel (b) shows the relative error of correlations (with respect to the fast variables) and FDR (with respect to responses) for several values of the filter constant T_w. The scale of the optimal value (as deduced from the theoretical argument) $T_w^\star = (\tau_0\tau_1^2)^{1/3}$ is marked by a dashed vertical line. Here $a_{12} = -0.5$, $a_{21} = -0.05$, $\varepsilon = 0.02$, $\tau_0 = 1$ and $\tau_1 = 1024$.

The above dynamics can be easily simulated with the standard Euler-Maruyama algorithm[3], and the proposed analysis can be applied to the numerically generated trajectories. The outcomes are shown in Figure S1. In panel (a) the solid lines represent the actual response functions, computed according to the definition, as in Eq. (1) of the main text (an average over a large number of realizations is considered). The dashed lines are obtained by naively applying the formula of generalized FDR valid for linear systems, Eq. (3) of the main text, to the dynamics of $\mathbf{x}$, which is neither Markovian nor linear. Finally, points are obtained by applying the generalized FDR to the properly filtered variables. This latter procedure is in good agreement with the results obtained by direct measure of response, as predicted by our analytical argument., while the naive analysis without filtering leads to quite misleading results. Panel (b) shows the relative error of correlation functions and GFDR as a function of T_w, the width of the Gaussian filter. As expected, the best results are obtained for T_w close to $(\tau_0\tau_1^2)^{1/3}$.

Inclusion of nonlinear terms

The above argument is valid when the interactions between the variables are linear; however one may study what happens when a nonlinear interaction term is added. It is indeed known that FDR is quite robust with respect to the addition of small nonlinear perturbations of the dynamics. In Fig. S2 we study model (S18) where

$$\dot{x}_1 = A_{11}x_1 + A_{12}x_2 \underline{-a x_2^3} + c_1 f_1(t) + \xi_1, \qquad \text{(S20)}$$

i.e. with the addition of the underlined nonlinear term.

As the figure shows, the presence of such nonlinear perturbation does not alter in a relevant way the ability of the filtering method to reproduce the actual response from long time series of data.

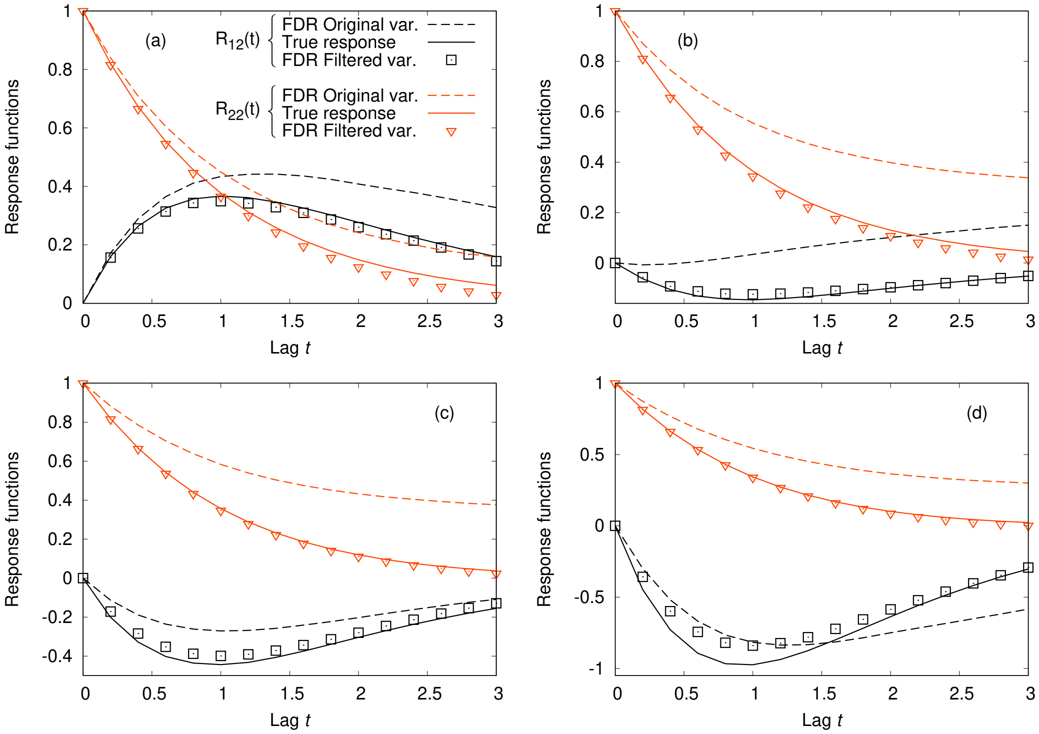

Figure S2. Same as Fig. S1(a), for model (S20) with different values of the coupling constant $\alpha = 0.1, 0.2, 0.4, 0.8$. Other parameters as in Fig. S1.

Lorenz ’63 forcing

A different kind of test consists in modifying the forcing with some less regular function. To this end, we use the first component of the Lorenz ’63 model

$$f(t) = x_L(t) \qquad \text{where} \qquad \begin{cases} \dot{x}_L = \frac{1}{\tau_1}\left[\sigma(y_L - x_L)\right] \\ \dot{y}_L = \frac{1}{\tau_1}\left[x_L(\rho - z_L) - y_L\right] \\ \dot{z}_L = \frac{1}{\tau_1}\left[x_L y_L - \beta z_L\right] \end{cases} \tag{S21}$$

with the usual choice of the variables $\sigma = 10$, $\beta = 8/3$, $\rho = 28$. The results are shown in Fig. S3.

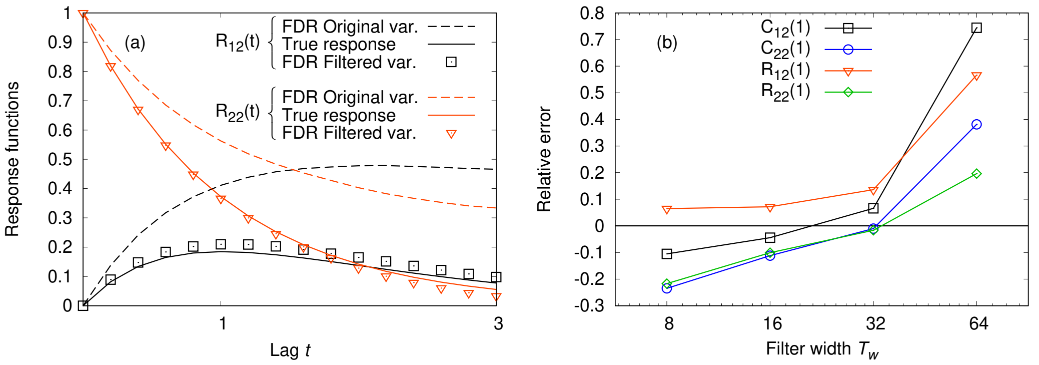

Figure S3. Same as Fig. S1, with a forcing described by Eq. (S21). In panel (a) the chosen value for the filter is $T = 32$. Other paramteres as in Fig. S1.

Transfer entropy: explicit formulation in the linear case

The definition of transfer entropy given in the main text [Eq. (18)] can be explicitely evaluated for the cases with linear dynamics[4,5]. Let us consider a linear N-dimensional Markov system $\mathbf{x}(t) = \{x_1(t), ..., x_N(t)\}$ with Gaussian statistics. The Shannon entropy associated to the corresponding stationary Gaussian distribution with covariance matrix $\Sigma_{\mathbf{x}}$ is, but for an irrelevant additive constant,

$$H_{\mathbf{x}} = \frac{1}{2} \ln |\Sigma_{\mathbf{x}}| \,, \tag{S22}$$

where $|M|$ stands for the determinant of the matrix M. It is also useful to remember that the covariance matrix of a conditioned Gaussian distribution verifies

$$\Sigma_{\mathbf{x}|\mathbf{y}} = \Sigma_{\mathbf{x},\mathbf{y}} \Sigma_{\mathbf{y}}^{-1} \Sigma_{\mathbf{x},\mathbf{y}}^{T} \,, \tag{S23}$$

where $\Sigma_{\mathbf{x},\mathbf{y}}$ is the covariance matrix of the joint distribution. Keeping this in mind, for the linear cases one can rewrite Eq. (18) of the main text as

$$TE_{1\to 2}(t) = \frac{1}{2} \ln \left(\frac{|\Sigma_{x_2(t)|x_2(0)}|}{|\Sigma_{x_2(t)|x_1(0),x_2(0)}|} \right) . \tag{S24}$$

Taking into account Eq. (S23), the above expression can be reduced into

$$TE_{1\to 2}(t) = \frac{1}{2} \ln \left(1 - \frac{\alpha_{21}(t)}{\alpha_{21}(t) - \beta_{21}(t)} \right) , \tag{S25}$$

with

$$\alpha_{21}(t) = [\Sigma_{22} C_{21}(t) - C_{22}(t) \Sigma_{21}]^2 \tag{S26a}$$

$$\beta_{21} = [\Sigma_{22}^2 - C_{22}(t)^2](\Sigma_{22}\Sigma_{11} - \Sigma_{21}^2) \,. \tag{S26b}$$

Further methodological remarks

To apply the proposed method to real situations, it is required that synchronised and equally time-spaced data are available, so that lagged cross-correlations can be suitably computed. To this aim, the original data series have to be interpolated, as discussed in the Method section of the main text. Of course this operation may introduce subtle sources of error. For instance, if the frequency of the interpolated data is larger than the original one, spurious correlations may be introduced, basically due to repeated use of the same data to generate the new points. It is thus important to carefully check that the interpolation procedure does not alter the results.

Figure 2 in the Methods section already shows that the temporal resolution of the temperature and CO_2 records is different and varies with time. Another way to further verify whether a non-homogeneously time-spaced dataset can be approximated by an interpolated series with a given lag relies on the analysis of the data population along the sample. The idea is to divide the total time interval into smaller segments and to count the number of data falling in each of them. The statistics of the data populations allows to determine how reliable the interpolation is, for a given value of the lag. If, for instance, most segments show a density of points which is lower than the inverse of the lag, this clearly means that in many parts of the dataset we are creating more points than the original ones. Note that this criterion is much stricter than simply considering the average lag between two data in the original series, as it takes into account the possibility of large fluctuations of the data density along the time series.

A graphical illustration of this analysis is shown in Fig. S4. From the figure it is clear that the $[CO_2]$ dataset is the most critical, as the amount of segments containing only few points is larger. To be more quantitative, about 80% of the considered intervals are populated by less than 20 points, meaning that the results obtained with a lag of 0.5 kyr (the first non-zero lag value in our plots) may be affected by the spurious correlations produced by the interpolation, as stated in the Method section of the main text. On the other hand, only 25% of the intervals contain less than 10 data points, meaning that the results at 1 kyr lag and larger are quite reliable under this respect.

To avoid potential issues emerging from the different resolution of the temperature and CO_2 signals, in the analysis we opted for degrading the resolution of the temperature signal to that of the CO_2 concentration (and vice-versa, in the few intervals

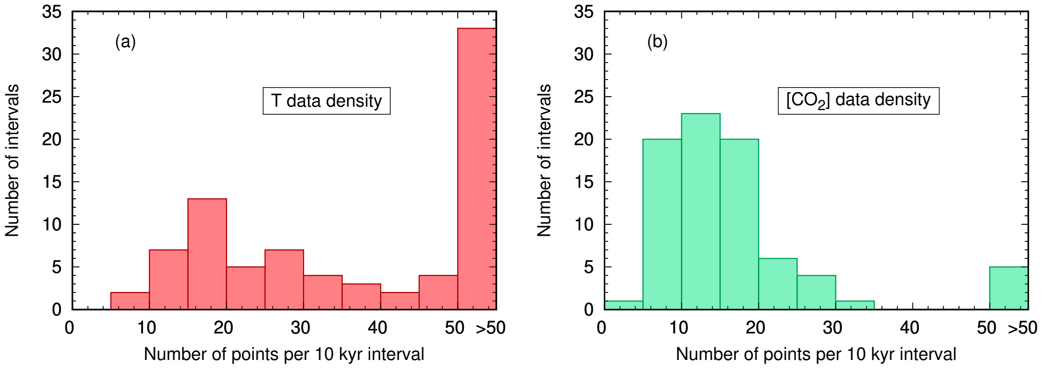

Figure S4. Density of paleoclimate data in the considered time series. For both T [panel (a)] and $[CO_2]$ [panel (b)] the dataset has been divided into 80 time intervals of 10 kyr duration, and the number of data points falling in each interval has been measured. The distribution of the data population indicates in this case that interpolations made with a frequency of 1 point per kyr, or less, are reliable since the number of intervals with lower data densities is relatively small for both samples.

where the temporal resolution of $[\mathrm{CO_2}]$ is larger than that of T), see Fig. 1 in the main text. Here, in Fig. S5 we show the results that would be obtained without such degradation of the resolution. In such case, the effect of $[\mathrm{CO_2}]$ on temperature becomes almost twice that of the opposite link, indicating that the estimated strength of the causal links can depend on many details, including differences in resolution. Clearly, such issues are amplified by the limited number of points available for the analysis. In any case, the main message of these results - that is, mutual causal relationships between $\mathrm{CO_2}$ concentration and temperature, which would be lost without high-pass filtering - does not change.

An interesting test is to repeat the analysis by considering only half of the dataset, to verify whether there is a temporal dependence of the causal relationships between temperature and $\mathrm{CO_2}$ concentration. Here, we consider either the first or the second half of the record, namely the data referring to either the first or the last 400 kyr. Of course, in this way the statistical uncertainty increases, as we are considering a more limited amount of data. Also in these cases, the high-pass filter allows to detect causal links on the scale of 1 kyr, which are hidden by the slow dynamics when performing analyses on unfiltered data. After applying the filtering procedure, the $[\mathrm{CO_2}] \to T$ causal link appears to dominate in the first 400 kyr, as shown in Fig. S6, while it becomes weaker than the reverse link in the last 400 ky, as shown in Fig. S7. This result can just be a statistical fluctuation generated by the limited amount of data, or it could point to a role of the temporal resolution in modulating the causal relationships of $\mathrm{CO_2}$ and T, or else it could reflect a true temporal variability in the strength of the causal links between the two variables. In any case, all these results confirm the presence of millenial-scale mutual causal relationships between $\mathrm{CO_2}$ and temperature, which could not be detected in the unfiltered record.

References

1. Falcioni, M., Isola, S. & Vulpiani, A. Correlation functions and relaxation properties in chaotic dynamics and statistical mechanics. *Phys. Lett. A* **144**, 341–346 (1990).

2. Gardiner, C. W. *Handbook of stochastic methods*, vol. 3 (springer Berlin, 1985).

3. Kloeden, P. E. & Platen, E. Stochastic differential equations. In *Numerical Solution of Stochastic Differential Equations*, 103–160 (Springer, 1992).

4. Sun, J., Taylor, D. & Bollt, E. M. Causal network inference by optimal causation entropy. *SIAM J. on Appl. Dyn. Syst.* **14**, 73–106 (2015).

5. Sarra, C., Baldovin, M. & Vulpiani, A. Response and flux of information in extended nonequilibrium dynamics. *Phys. Rev. E* **104**, 024116, DOI: 10.1103/PhysRevE.104.024116 (2021).

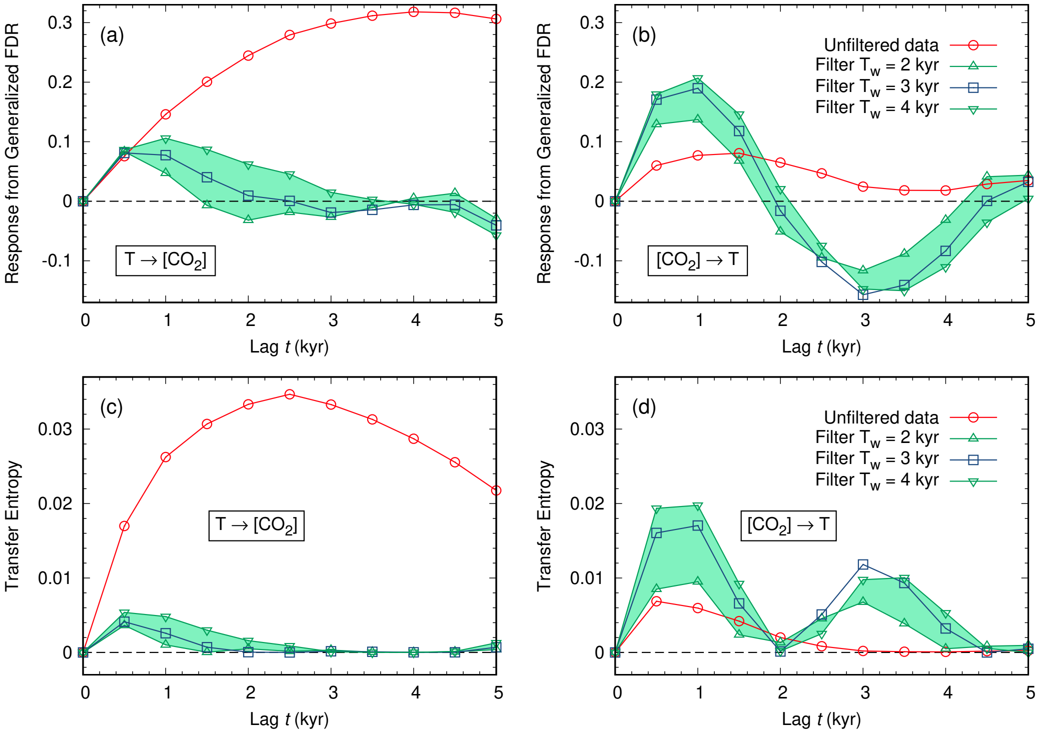

Figure S5. Analysis of the mutual influence between temperature T and CO_2 concentration using the data with undegraded temporal resolution. Panels (a) and (b) show the response function, computed according to the Generalized FDR. Panel (a) refers to the effect of T on $[CO_2]$, while panel (b) shows the opposite relation. Red circles represent the results of a direct application of Eq. (3) of the main text to raw data, apparently suggesting that $T \rightarrow [CO_2]$ is much stronger than $[CO_2] \rightarrow T$. The response computed from data filtered by a $T_w = 3$ kyr window (blue squares) instead indicates that the impact of $[CO_2]$ on T becomes larger. The result is robust with respect to T_w variations by one kyr (green up/down triangles). A similar analysis, where TE is computed instead of the generalised FDR, is shown in Panels (c) and (d). Here, we have considered the data with their original temporal resolution, which is different for temperature and CO_2 concentration.

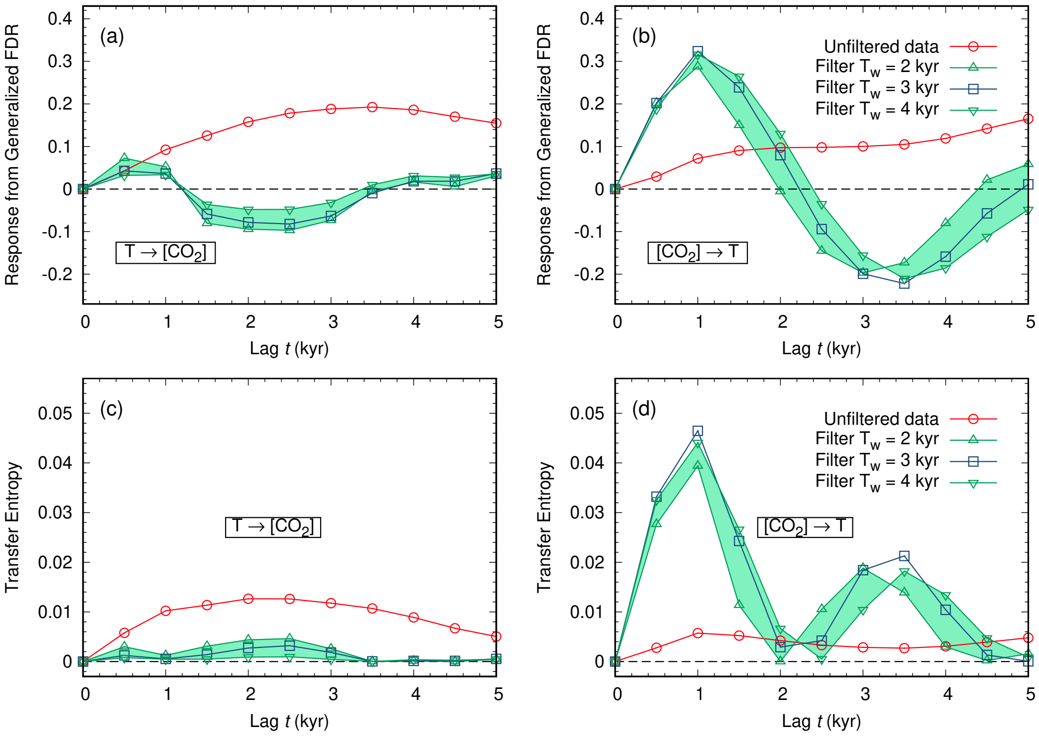

Figure S6. Analysis of the causal relations between T and $[CO_2]$ from paleoclimate data, using a spline interpolation and considering only the first 400 kyr of the record. As before, the cross terms of the generalized FDR [panels (a) and (b)], as well as the corresponding transfer entropies [panels (c) and (d)] are shown. Each quantity has been computed before and after the high-pass filtering procedure of the data series.

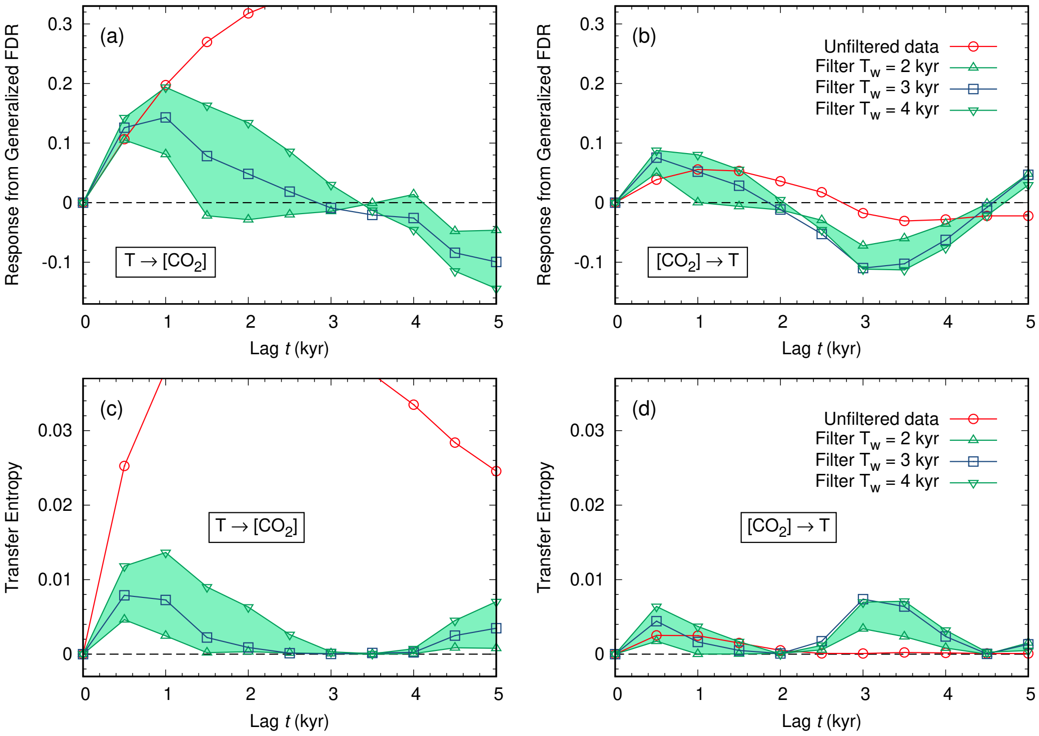

Figure S7. Analysis of the causal relations between T and $[CO_2]$ from paleoclimate data, using a spline interpolation and considering only the last 400 kyr. As before, the cross terms of the generalized FDR [panels (a) and (b)], as well as the corresponding transfer entropies [panels (c) and (d)] are shown. Each quantity has been computed before and after the high-pass filtering procedure of the data series.
